# Oligogalacturonic acids promote tomato fruit ripening through the regulation of 1-aminocyclopropane-1-carboxylic acid synthesis at the transcriptional and post-translational levels

**DOI:** 10.1186/s12870-015-0634-y

**Published:** 2016-01-09

**Authors:** Yingxuan Ma, Leilei Zhou, Zhichao Wang, Jianting Chen, Guiqin Qu

**Affiliations:** College of Food Science and Nutritional Engineering, China Agricultural University, Beijing, 100083 People’s Republic of China

**Keywords:** ACC synthase 2, Ethylene, Oligogalacturonic acids, Protein phosphorylation, Tomato fruit ripening

## Abstract

**Background:**

Oligogalacturonic acids (OGs) are oligomers of alpha-1,4-linked galacturonosyl residues that are released from cell walls by the hydrolysis of polygalacturonic acids upon fruit ripening and under abiotic/biotic stress. OGs may induce ethylene production and fruit ripening, however, the mechanism(s) behind these processes is unknown.

**Results:**

Tomato cultivar ‘Ailsa Craig’ (AC) and mutant *Neverripe, ripening inhibitor, non-ripening*, and *colorless non-ripening* fruits were treated with OGs at different stages. Only AC fruits at mature green stage 1 showed an advanced ripening phenomenon, although transient ethylene production was detected in all of the tomato fruits. Ethylene synthesis genes *LeACS2* and *LeACO1* were rapidly up-regulated, and the phosphorylated LeACS2 protein was detected after OGs treatment. Protein kinase/phosphatase inhibitors significantly affected the ripening process induced by the OGs. As a potential receptor of OGs, LeWAKL2 was also up-regulated in their presence.

**Conclusions:**

We demonstrated that OGs promoted tomato fruit ripening by inducing ethylene synthesis through the regulation of LeACS2 at transcriptional and post-translational levels.

**Electronic supplementary material:**

The online version of this article (doi:10.1186/s12870-015-0634-y) contains supplementary material, which is available to authorized users.

## Background

The ripening of fleshy fruits causes complex biochemical changes during the transformation from the developmental program to the ripening process, which is regulated by hundreds to thousands of genes [1–3]. Ethylene is essential for the initiation and completion of tomato (*Solanum lycopersicum* L.) fruit ripening [1, 4, 5]. Aminocyclopropane-1-carboxylic acid synthase (ACC synthase, ACS; EC4.4.1.14) and ACC oxidase (ACO; EC1.3.3.6) are responsible for ethylene biosynthesis, which catalyze the conversion of S-adenosyl-methionine to ACC and ACC to ethylene, respectively [6]. There are many innate elicitors that can induce ethylene production in plants, including abscisic acid (ABA), which can phosphorylate the C-terminus of AtACS6 by activating *Arabidopsis thaliana*’s calcium-dependent protein kinases *AtCDPK4* and *AtCDPK11* [7], and auxin, which can inhibit ABA-induced stomatal closure by promoting ethylene production [8]. Additionally, the cell wall degradation products released during the regular fruit ripening process can also induce ethylene production, playing an important role in the complex process of fruit ripening [9, 10].

Oligogalacturonic acids (OGs) are oligomers of alpha-1,4-linked galacturonosyl residues released from the cell wall by the hydrolysis of polygalacturonic acids [11] upon microbial infection [12] and mechanical damage [13], as well as during fruit ripening [14]. As a plant innate elicitor, OGs can induce a series of plant responses [15], including ethylene synthesis [16], the inhibition of auxin action [17, 18], the accumulation of phytoalexins [11, 19] and callose, and the production of reactive oxygen species [20] and nitric oxide [21]. Previous studies demonstrated the endogenous accumulation of pectin-derived oligosaccharides, including OGs, in tomato tissues that were just beginning to ripen and these promoted a short increase of ethylene production in MG tomato pericarp discs [22]. Also, the pectin breakdown products of tomato fruits can be induced by pathogen-related enzyme action [23]. These studies indicate that endogenous pectin-derived oligosaccharides exist and function in the normal course of ripening and disease defense in tomatoes. Early studies suggested that OGs could promote ethylene biosynthesis in tomato fruits or discs, and in pear cell suspensions [9, 10, 24]. A mixture of small-sized OGs elicited ethylene production in tomato plants as a response to wounding [25]. OGs with four to six degrees of polymerization (DP) were shown to be more effective in ethylene promotion through their ability to induce the expression of the *LeACO1* gene [16, 25]. Although the OGs’ ability to enhance ethylene production has been shown, the mechanisms behind this capability have not been elaborated and whether the OGs impact the fruit ripening progress remains unclear.

Recent studies found that OGs could mediate cell wall signal transduction and are recognized by wall-associated kinases and kinase-like proteins (WAKs and WAKLs, respectively), which contain an extracellular domain, a transmembrane domain, and a cytoplasmic kinase domain [26]. In *Arabidopsis*, AtWAK1 interacts with cell wall pectins in a calcium-induced conformation and is an OGs receptor [27, 28], OGs can affect many plant developmental and stress responses by activating the mitogen-activated protein kinases (MAPKs) in a WAK2-dependent manner [29, 30]. Activated MAPKs can regulate a series of phytohormones, including salicylic acid, jasmonic acid, and ethylene, which extensively modulate plant growth, development, and stress/defense responses [31, 32]. Several ACS proteins can be regulated by MAPKs through phosphorylation and dephosphorylation [33, 34]. The phosphorylation of AtACS2 and AtACS6 by MAPK6 led to the accumulation of the ACS protein and elevated levels of cellular ACS activity, promoting ethylene production [35, 36]. Calcium-dependent protein kinases (CDPKs) are also implicated in ACS regulation [34]. In tomato, phosphorylation by MAPK and CDPK are both required to promote LeACS2 stability in the wounded tomato pericarp, and the phosphorylation/dephosphorylation of LeACS2 regulates its turnover upstream of the ubiquitin-26S-proteasome degradation pathway [37, 38].

In this study, we found that OGs could promote the ripening of the tomato cultivar ‘Ailsa Craig’ (AC) fruits at the mature green 1 (MG 1) stage by inducing ethylene biosynthesis. Additionally, the transcriptional levels of *LeACS2* and *LeACO1* were up-regulated in the presence of OGs. OGs also induce the phosphorylation of *LeACS2* at Ser-460. These results demonstrated that OGs induced ethylene biosynthesis at transcriptional and post-translational levels, and then promoted the ripening of tomato fruits. In addition, as a candidate OGs receptor, LeWAKL2 was affected by OGs, which remains to be studied further.

## Methods

### OGs preparation and separation for different DP

OGs mixture with different degrees of polymerization were prepared from polygalacturonic acid (PGA, Sigma 81325, the purity > 95 %) according to previous studies [16] with adjustment. 1 g PGA was dissolved in 100 mL 0.1 M pH 4.4 sodium acetate, PGA was first incubated with 100 μL pectin methylesterase (PME, EC 3.1.1.11, extracted from ripe tomato fruits) at 37 °C for 2 h with shaking to remove the methyl residues, the solution was heated to 100 °C for 5 min to inactivation the PME, 5 U polygalacturonase (PG, EC 3.2.1.15, Sigma 17389, from *Aspergillus niger*, enzymatic activity > 1 U/mg) was then added and incubated for 1 h at 37 °C, finally incubated at 100 °C for 10 min.

We used anion exchange chromatography on a QAE-Sephadex A-25 matrix (Pharmacia, 2.5 × 160 cm) to separate OGs with different DP, after equilibrated with 0.125 M imidazole HCL buffer pH 7.0 for 200 mL [19], pH 7.0 imidazole HCL of 0.2 M, 0.35 M, 0.5 M, 0.65 M, 0.8 M, 0.9 M, 1.0 M were used to elute the OGs with 500 mL for each concentration. 9 mL elution was collected for each pool, thin-layer chromatography (TLC) and total uronic acids content was detected with m-phenyl phenol method [39] (Additional file 4), pools with same DP were combined and desalted with Sephadex G-25 matrix (Pharmacia, 2.5 × 160 cm). Separation results were further analyzed by matrix-assisted laser desorption ionization-mass spectrum (MALDI-MS) as shown in Additional file 4 according to Simpson et al. [16].

The OGs we used in experiments were mixture of all the OG individuals according to our pre-experiment that mixed OG with all different DP had similar function to OGs with mixed DP ≥ 9 (see Additional file 5). The sizes of mixed OG were shown in Additional file 4A, and the control solution was prepared with the same procedure without PGA.

### Plant materials, growth conditions and treatment methods

Tomato (*Solanum lycopersicum*) wild type plant AC, mutants of *Nr*, *rin*, *nor* and *Cnr* were grown in the green-house at Xiaotangshan Vegetable Planting Base, Beijing under standard conditions (25/20 °C). AC and mutants seeds were kindly provided by Dr. Jim Giovannoni (Boyce Thompson Institute for Plant Research, Ithaca, NY 14853, USA). Fruits at different stages were picked according to the days after pollination (DAP) described by previous studies [40] kept with carpopodium. MG1 was 39 DAP. All the fruits were washed with ddH_2_O and dried by airing, balanced their temperature to 25 °C overnight before the measurement of ethylene production. The ethylene production was then detected for further classification of AC MG fruits as described by previous studies [41], MG 1 stage had an ethylene production of 0–0.1 nL · g^−1^FW · h^−1^, MG 2 was 0.1–0.4 nL · g^−1^FW · h^−1^ and MG 3 was 0.4–0.7 nL · g^−1^FW · h^−1^.

Fruits’ carpopodium were discarded just before treatment, total 10 μL · g^−1^FW of 10 μM K252a in 0.2 % dimethylsulfoxide (DMSO) or 1 μM OA in 0.2 % DMSO were added at the cutting point, K252a or OA were absorbed through vacuum infiltration under −0.02 MPa for 2 min and then balanced 10 min for further assimilation. After one hour, fruits were treated with 1 g/L OGs or the control solution under the same condition unless the fruits were immersed in the container to absorb the OGs. Surplus solution on the surface was blotted with paper, and the fruit pericarps on the equator were frozen in liquid nitrogen at 1, 2, 3, 5, 8, 12 and 24 h after treatment for short-term variation detection. Samples were also froze every day after treatment until 11 days to detect the ethylene and color changes. All samples were stored at −80 °C until use.

Fruit pericarp discs were used to detect the short-term ethylene production of AC and mutant fruits. The fruit pericarp discs with 1 cm diameter were placed on wet filter paper, and put in a 100 % humidity ventilated climate box overnight at 25 °C. Pericarp discs were incubated with 1 g/L OGs or the control solution at 25 °C for 2 h, redundant OGs on surface were soaked up by filter paper and then several discs were selected to determine ethylene production. Remained pericarp discs were placed in the climate box until for ethylene detection. We detected ethylene production at 0 h, 1 h, 3 h and 6 h after treatment with four repeats at each time point.

### Ethylene measurement

For fruit ethylene detection, each fruit was placed in a gas tight 300 mL container at 25 °C for 1 h, and 1 mL gas sample was analyzed using gas chromatograph (GC) equipped with a flame ionization detector (Shimadzu, Japan, http://www.shimadzu.com/) to detect ethylene production as described previously [42]. For the detection of pericarp discs ethylene production, each four discs were placed in a 12 mL bottle for 1 h, 1 mL gas sample was analyzed as described above.

### RNA extraction and real-time PCR

Fruit total RNA were extracted using QIAGEN RNA extraction kit (http://www.qiagen.com). cDNAs were synthesized from 1 μg of total RNA using Transgen one-step gDNA removal and cDNA synthesis supermix (http://www.transgen.com.cn). For quantitative RT-PCR analysis, a Bio-Rad CFX96 real-time PCR detection system was used in standard mode with SYBR Green Supermix (Transgene, http://www.transgene.com.cn). Products were verified by melting curve analysis, and mRNA abundance was analyzed using the relative standard curve method with normalization to *LeActin*. All primer sequences are listed in Additional file 6. Gene IDs used for RT-PCR and western blot in this work are provided in Additional file 7.

### Antibody preparation

The antibody of phospho-LeACS2 was prepared by Beijing Protein Innovation (www.proteomics.org.cn) according to previous studies [37]. Phospho-peptide (NH_2_-CKNNLRLpSFSKRMY-OH) was synthesized with the phosphorylation site at Ser-460 [38], corresponding to the Lys-454 to Tyr-466 sequence. A rabbit was immunized with phospho-peptide conjugated bovine serum albumin by multiple intradermal injections. The rabbit’s serum was applied to a non-phospho-peptide conjugated column and the flow-through fraction was applied to the phospho-peptide column. Bound IgG was eluted with 0.1 M glycine-HCL (pH 2.5) and immediately adjusted to pH 8.0.

The antibody for detection of LeWAKL2 was prepared with antigen area 532 ~ 703 aa. Rabbit polyclonal antibody was prepared by Beijing Protein Innovation (www.proteomics.com.cn).

### Protein extraction and denaturing conditions

Fruit samples about 0.5 g were homogenized with 1.5 mL 10 % tri-chloro acetic acid in acetone, centrifuged at 16,000 ***g*** for 3 min at 4 °C, the precipitation was then mixed with 0.1 M ammonium acetate in 80 % MeOH, centrifuged at 16,000 ***g*** for 3 min at 4 °C. The supernatant was discarded and the sediment was washed by 80 % acetone, after homogenized with 1.5 mL phenol/SDS solution [Tris-phenol, pH 8.0; SDS buffer (30 % sucrose, 2 % SDS, 0.2 M Tris pH 8.0, 5 % β-mercaptoethanol); v:v = 1:1], 0.1 M ammonium acetate in 80 % MeOH was added and incubated at −20 °C overnight. Centrifuged at 16,000 ***g*** for 3 min at 4 °C, the pellet was washed with MeOH and 80 % acetone successively, after air dry, the proteins were suspended by 100 μL SDS buffer (0.5 M Tris pH 7.0, 1.4 % SDS). Protein concentration was determined by the method of Bradford [44] using bovine serum albumin as standard. Proteins were dissolved in 5 × sample loading buffer [125 mM Tris-HCl (pH 6.8), 2 % SDS, 2 % β-mercaptoethanol and 0.1 % bromophenol blue], boiling at 100 °C for 3 min and instantly inserted into the ice.

### Western blot analysis

Proteins were separated using SDS-PAGE (8 % acrylamide gels) and blotted onto nitrocellulose membranes (0.45 μm; Whatman, http://www.whatman.com). The membrane was blocked with 5 % dried skimmed milk and 0.05 % Tween 20 in Tris-buffered saline [20 mM Tris-HCl (pH 7.5), 150 mM NaCl] for 2 h at room temperature. Purified anti-phosphorylated-LeACS2 or LeWAKL2 antibody was incubated overnight at 4 °C. The membrane was washed with 0.05 % Tween 20 in Tris-buffered saline and then reacted with horseradish peroxidase-conjugated goat anti-rabbit IgG (EASYBIO, http://www.bioeasytech.com) at a dilution of 1:10,000. Western chemiluminescent HRP substrate was bought from Millipore Corporation. The relative intensities of bands were quantified by Adobe Photoshop CC.

## Results

### OG-induced tomato fruit ripening is developmentally regulated

**Fig. 1:**
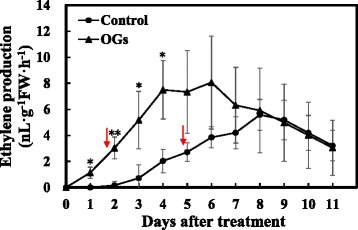
Ethylene production of AC MG1 fruits treated with or without OGs. Tomato fruits were placed in a ventilated and temperature constant room at 25 °C and treated with 1 g/L OGs or the control solution. Each fruit was put in a 300 mL air tight container at 25 °C for 1 h and 1 mL gas was extracted to detect ethylene content. Vertical bars indicate the *SD* (*n* = 12). Asterisks indicate statistically significant differences compared with the control group (*P < 0.05; **P < 0.01, Student’s *t*-test). The red arrow indicates the ethylene production of 3 nL · g^−1^FW · h^−1^ which corresponds to the breaker stage of tomato fruit on the plant

Wild-type AC tomato fruits were treated at MG 1, 2, and 3 stages with OGs prepared as previously reported [16]. Ethylene production was detected every day after treatment, and fruits were photographed. Only MG1 fruits showed an accelerated ripening process, and the differences were found in two aspects of MG 1 fruits. Ethylene synthesis was accelerated to 3 nL · g^−1^FW · h^−1^ by 2 days after treatment, approximately 3 days earlier than the control group (Fig. 1). Furthermore, the maximum rate of ethylene biosynthesis was enhanced. Ethylene accumulated to ~8 nL · g^−1^FW · h^−1^ by 4 days after treatment and stayed at this high value until 6 days, when it started to slowly and steadily decrease. In the control fruits, a relatively lower ethylene synthesis rate was detected until 8 days after treatment. The rates were similar in both groups after 8 days. The OG-treated fruit’s ethylene synthesis rates were not significantly different than those treated with control solution at other stages (Additional file 1).

**Fig. 2:**
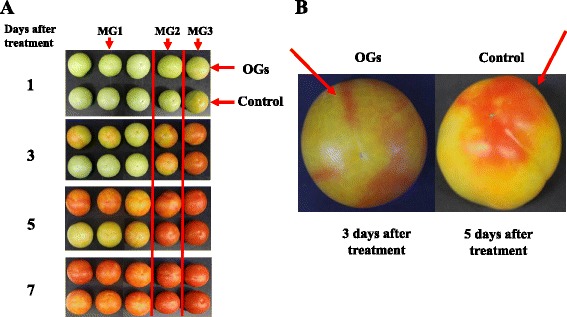
Color changes of AC fruits treated with or without OGs. **a** MG 1, MG 2 and MG 3 stage AC fruits were treated with 1 g/L OGs or the control solution through vacuum infiltration. Photos taken 1, 3, 5 and 7 days after conduction. **b** Detail color changes of MG 1 stage fruits, 3 or 5 days after treatment with OGs or the control solution respectively, red arrow indicates the position that had first turned red

Accompanying the ethylene production, OG-treated AC MG 1 stage fruits showed an accelerated color change that occurred approximately 2 days earlier than the color change in the control group (Fig. 2a). No alterations in the timing of the color changes were found on OG-treated MG 2 or MG 3 stage fruits (Fig. 2a). The emerging pattern of redness in OG-treated fruits was found linearly on the fruits’ surfaces, not from the top of the fruit as in controls (Fig. 2b). We further found that the red areas on the fruits’ surfaces corresponded to the septum where OGs mainly accumulated when they were applied to fruit using vacuum infiltration through the joint between fruit and carpopodium.

The data demonstrated that OGs could induce tomato fruit’s ethylene production and promoted the MG1 fruit ripening process. The results using different fruit stages suggested that the tomato fruit’s response to the OGs’ induced ripening was developmentally regulated.

### Ethylene perception and signaling pathways are necessary for OG-promoted fruit ripening

**Fig. 3:**
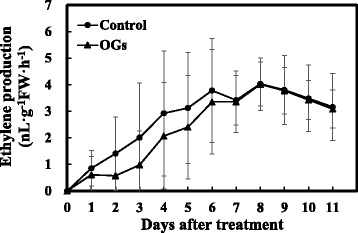
Ethylene production of *Nr* fruits treated with or without OGs. Each fruit was put in a 300 mL air tight container at 25 °C for 1 h. 1 mL gas was extracted to detect ethylene content. Vertical bars indicate the *SD* (*n* = 12)

To identify whether the ethylene perception and/or signaling pathways participated in OGs’ promotion of fruit ripening, tomato fruit mutants *Neverripe (Nr), ripening inhibitor (rin), non-ripening (nor)*, and *colorless non-ripening (Cnr)* were treated with OGs or the control solution. The MG *Nr* fruits had continuously increasing ethylene production levels after treatment, but no obvious differences were found between the treatment and control groups (Fig. 3). As *Nr* is an ethylene receptor mutant, resulting in the inhibition of ethylene perception [39], this result indicated that the ripening process stimulated by the OGs relied on ethylene perception.

The *rin*, *nor*, and *Cnr* mutants all had drastically reduced ethylene production levels and the ripening process could not be restored by an exposure to exogenous ethylene, indicating a functional defect in ethylene signaling [34]. We treated these mutants with OGs to test whether the ethylene signaling pathway was necessary for OG-induced ethylene production as well as fruit ripening. No obvious differences were found among the *rin*, *nor*, and *Cnr* fruit ripening processes with or without OGs treatments (Additional file 2), indicating that the ethylene signal transduction pathway was necessary for OG-induced tomato fruit ripening.

In our experiments on the OG-promoted tomato fruit ripening, the ethylene production was measured starting 1 day after OGs treatment. Campbell et al. found that ethylene production in pectic oligomers treated tomato fruit discs showed a transient increase [9, 10]. Therefore, to further examine whether a short-term boost in ethylene production existed in OG-treated tomato-ripening mutant fruits, we measured the ethylene production of *Nr*, *rin*, *nor*, and *Cnr* fruit discs after OGs treatment. A transient ethylene production was observed in all of the mutant fruits after OGs treatment (Additional file 3). These results indicated that OG-induced transient ethylene production is not blocked by the lack of transcription factors RIN, NOR, CNR or ethylene receptor NR.

### OG-regulated expression patterns of ethylene synthesis-related genes

**Fig. 4:**
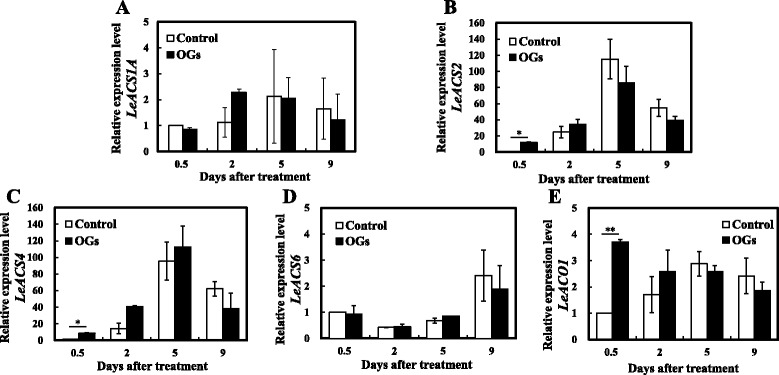
Relative expression levels of ethylene synthesis-related genes. *LeACS1A* (**a**), *LeACS2* (**b**), *LeACS4* (**c**), *LeACS6* (**d**) and *LeACO1* (**e**) gene expression values were presented relative to the *Actin*. Vertical bars indicate the *SD* (*n* = 3). Asterisks indicate statistically significant differences compared with the control group (*P < 0.05; **P < 0.01, Student’s *t*- test)

To explore whether OGs affect ethylene synthesis-related gene expression levels, we measured the long term expression levels of *LeACS1A*, *LeACS2*, *LeACS4*, *LeACS6*, and *LeACO1* after OGs treatment (Fig. 4). *LeACS2*, *LeACS4*, and *LeACO1* were up-regulated by 12 h after treatment with OGs, but no significant differences were found after the second day. *LeACS1A* and *LeACS6* were not obviously changed by the OGs and both groups stayed at low levels. Because of the effects of OGs on ethylene production and the distinct changes in the transcriptional levels of related genes within the first day, obtaining more detailed ethylene production and gene expression profiles during the first 24 h was necessary.

**Fig. 5:**
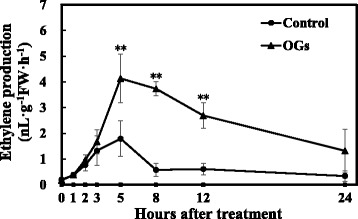
Transient ethylene production of AC MG1 fruits with or without OGs treatment. Vertical bars indicate the SD (*n* = 6). Asterisks indicate statistically significant differences compared with the control group (*P < 0.05; **P < 0.01, Student’s *t*-test)

**Fig. 6:**
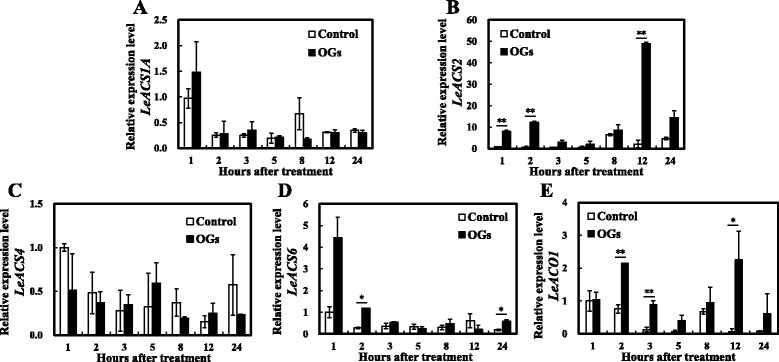
Relative expression levels of ethylene synthesis-related genes within 24 h. *LeACS1A* (**a**), *LeACS2* (**b**), *LeACS4* (**c**), *LeACS6* (**d**) and *LeACO1* (**e**) gene expression values were presented relative to the *Actin*. Vertical bars indicate the *SD* (*n* = 3). Asterisks indicate statistically significant differences compared with the control group (*P < 0.05; **P < 0.01, Student’s *t*-test)

We first measured the ethylene production of AC fruits during the 24 h after treatment with OGs or the control solution. As shown in Fig. 5, OGs treatment induced a significant burst of ethylene production compared with control fruits by 5 h, 8 h and 12 h. For the gene expression levels, *LeACS2* was induced by OGs 1 h after treatment, although this enhancement was only sustained for 2 h. Then, it increased again from the 8 h, having a ~50 fold increase over the control group at 12 h after treatment (Fig. 6b). A similar expression pattern was found for *LeACO1* (Fig. 6e), although the change was not as significant as that of *LeACS2. LeACS6* was also induced by OGs 1 h after treatment, however this effect was transient and not as strong as *LeACS2* (Fig. 6d). No obvious differences were found between the treatment groups for *LeACS1A* and *LeACS4,* and they all decreased rapidly from 1 h after treatment (Fig. 6a, c). Combined the results of ethylene production and ethylene synthesis-related genes expression indicated that *LeACS2* and *LeACO1* are the two related genes involved in transcriptional regulation of OGs induced transient ethylene production.

### OG-induced ethylene synthesis is phosphorylation regulated and LeACS2 is phosphorylated after OGs treatment

**Fig. 7:**
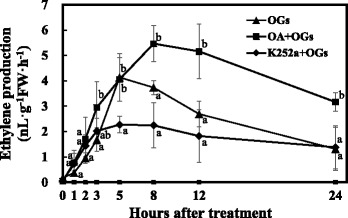
Protein kinase/phosphatase inhibitors regulate OGs’ effect on ethylene synthesis. AC MG1 fruits were treated with 10 μM K252a in 0.2 % DMSO, 1 μM OA in 0.2 % DMSO or 0.2 % DMSO, respectively, 1 h before treatment with OGs. Vertical bars indicate the *SD* (*n* = 6). Significant differences of four groups at each time point were indicated by different letters (Tukey’s HSD, *P* < 0.05)

LeACS2 turnover was adjusted by phosphorylation/dephosphorylation and occupied an important position in ethylene synthesis. Phosphorylated LeACS2 at Ser-460 proved to be more stable and boosted ethylene synthesis [38]. To identify whether phosphorylation/dephosphorylation process involved in OGs induced ethylene production, fruits were treated with protein kinase/phosphatase inhibitors 1 h before treatment with OGs. Ethylene production was detected after treatment as shown in Fig. 7. The protein kinase inhibitor K252a apparently inhibited the OGs’ promotion of ethylene synthesis by 5 h after treatment. In contrast, the protein phosphatase inhibitor okadaic acid (OA) enhanced the ethylene production induced by the OGs and the differences were significant by 8 h, 12 h and 24 h after treatment compared with OGs treatment alone. This suggested that phosphorylation/dephosphorylation affected the ethylene synthesis induced by the OGs.

**Fig. 8:**
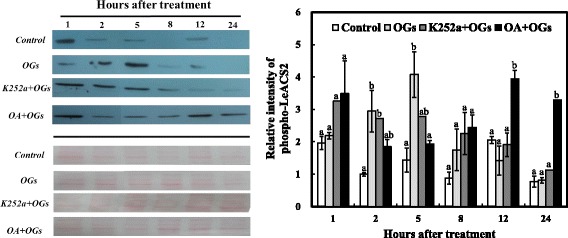
Time-course analysis of phosphorylated LeACS2 treated with or without OGs. Proteins were extracted from samples the same for gene expression detection. Total 25 μg protein were loaded on each line to separate through 8 % SDS-PAGE gel, anti-phosphorylated-LeACS2 was incubated with membrane overnight at 4 °C. Ponceaux dyeing was used to verify the protein amount. The relative intensities of phosphorylated-LeACS2 were quantified with Adobe Photoshop CC. We used the second hour protein band intensity of the control group as standard. The experiment was repeated in triplicate. A representative gel is shown. Significant differences of four groups at each time point were indicated by different letters (Tukey’s HSD, *P* < 0.05)

Additionally, we selected LeACS2 as a target to detect the phosphorylation level using a western blot analysis with anti-phosphorylated LeACS2 at Ser-460 after OGs treatment. OGs induced the accumulation of phosphorylated LeACS2 from 1 h, with its highest value occurring at 5 h, and little phosphorylated LeACS2 was found later (Fig. 8). Protein kinase inhibitor K252a reduced LeACS2 gradually after treatment, although the value detected was higher at the first hour compared with samples treated with OGs alone. Phosphorylated LeACS2 was also accumulated at 1 h when pretreated with OA, but this effect rapidly decreased and did not return until 12 h, showing the long-term effects of OA on OGs treatment.

### LeWAKL2 was induced by OGs as a candidate receptor

**Fig. 9:**
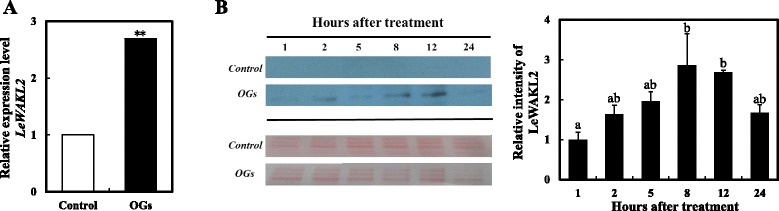
Gene expression and protein variation of LeWAKL2. **a** Gene expression levels of *LeWAKL2* by 2 h after OGs treatment, and the values were presented relative to the *Actin*. The experiment was repeated in triplicate, and the asterisks indicate statistically significant differences compared with the control group (*P < 0.05; **P < 0.01, Student’s *t*-test). **b** Protein accumulation of LeWAKL2. Total 25 μg protein were loaded on each line to separate through 8 % SDS-PAGE gel, and anti-LeWAKL2 was incubated with membrane overnight at 4 °C. The relative intensities of LeWAKL2 were quantified with Adobe Photoshop CC for OGs treated group. We used the first hour LeWAKL2 protein band intensity as standard since no signals were detected for the control group. Ponceaux dyeing was used to verify the protein amount. The experiment was repeated in triplicate. A representative gel is shown. Significant differences of four groups at each time point were indicated by different letters (Tukey’s HSD, *P* < 0.05)

The WAKs/WAKLs family members were considered candidate receptors of the OGs as shown in *A. thaliana* and were involved in signal transduction after OGs treatment [28, 30, 45, 46]. Here, we found that *LeWAKL2* was induced by OGs to a level approximately three-fold greater than that in the control fruits by 2 h after treatment (Fig. 9a). We also performed a western blot analysis with anti-LeWAKL2 (532–703 aa) to detect the protein level after treatment. LeWAKL2 continued to accumulate after treatment, reaching its highest value at 8 h, and no LeWAKL2 signal was detected in fruits treated with the control solution (Fig. 9b).

## Discussion

OGs, as plant elicitors, can regulate ethylene, auxin and other phytohormones in plant development and innate defenses [16–18]. Our experiments confirmed that exogenous OGs could induce ethylene production in the short and long term, and accelerated fruit ripening, which were consistent with a previous study using fruit pericarp discs [10]. Here we showed that this acceleration of fruit ripening was developmentally regulated and that MG 1 stage fruits showed obvious phenotypes (Fig. 2). We speculated that the transient ethylene production played critical roles in the acceleration of tomato fruit ripening and the effects of OGs on MG2/3 fruits might be masked as the fruit matured and produced more ethylene. Cutillas-Iturralde et al have shown that ethylene was induced by exogenous application of xyloglucan-derived oligosaccharides in persimmon (*Diospyros kaki L.*) and the later harvested fruits showed more evident ethylene production after treatment [47]. Although persimmon fruits synthesized lower ethylene compared with tomato fruits, this phenomenon strengthened our idea that oligosaccharides’ function on fruit ethylene synthesis correlated with fruit development stage.

The system-2 ethylene synthesis was initiated at the onset of climacteric fruit ripening, and autocatalytic ethylene synthesis has a pivotal role in the system-2 ethylene synthesis, as well as the ripening process [48, 49]. Whether the OGs induced acceleration of fruit ripening required ethylene signal transduction was not clear. In our experiments, we found that fruit pericarp discs of AC, *Nr*, *rin*, *nor*, and *Cnr* all showed obvious short term ethylene bursts after being treated with OGs (Additional file 3). We considered that the OG-induced transient ethylene production was similar to the ethylene production initiated by wounding, which can be elicited rapidly [50]. However, our data indicated that long term ethylene synthesis and the ripening process of mutant fruits with defective climacteric respiration in ethylene biosynthesis were all not affected by OGs (Fig. 3, Additional file 2). Therefore, we speculated that OGs’ functions in fruit ripening required system-2 ethylene synthesis and the autocatalytic regulation by the ethylene signaling pathway. Additionally, previous studies found that exogenous ABA could accelerate the ripening process of MG tomato fruits and this appears to require ethylene production because 1-MCP could fully block ABA’s effect on fruit ripening and softening [51]. Thus, autocatalytic ethylene synthesis is required for both OG- and ABA-triggered fruit ripening.

The transcriptional and post-translational regulation of ethylene synthesis proved pivotal for plant development and ripening [34, 52–54]. Previous studies demonstrated that wound- and ABA-induced ethylene synthesis mostly depended on post-transcriptional regulation [7, 38, 55]. However, we found that the regulation of OGs on ethylene production was at both the transcriptional and post-translational levels. At the transcriptional level, *LeACS2* and *LeACO1* were induced twice after OGs treatment (Fig. 6), which was consistent with previous researches in which *AtACS6, AtACO*, *AtERF1*, and *AtERF5* were all induced by OGs treated *A. thaliana* cell suspensions [56]. Further, we found that phospho-LeACS2 was accumulated after the treatment, with its highest value occurring after 5 h. K252a decreased protein accumulation and OA enhanced protein accumulation, especially after the 12 h (Fig. 7). Although K252a and OA are not specific protein kinase inhibitors, previous studies demonstrated that K252a can block the phosphorylation of LeACS2 by MAPK and CDPK [38]. Similar results were found in rose flowers [57].

The WAK/WAKL family are receptor-like kinases linked to the cell wall and contain a cytoplasmic protein kinase domain [40]. AtWAK1 has been demonstrated to be an OGs receptor using a domain swap approach [28], and that AtWAK2 functioned upstream of MAPK during stress [29, 30]. Here, we found that LeWAKL2 production was promoted by the OGs at the transcriptional and post-translational levels (Fig. 9). Previous studies found that the *LeWAKL2* gene can be increased early in tomato roots and in cell suspensions challenged with *Orobanche ramosa* [58]. Thus, LeWAKL2 may be an OGs receptor, passing signals from the OGs and activating specific LeMAPKs or LeCDPKs, although further researches are needed to elucidate whether OGs can bind LeWAKs/WAKLs or how OG-induced ethylene production is regulated by LeWAKs or LeWAKLs.

## Conclusions

In this paper, we investigated the OGs’ acceleration of tomato fruit ripening and explained the detailed regulation of ethylene synthesis. OGs treatments of different fruit stages indicated that the OGs’ effect on ripening is developmentally regulated and that MG1 stage tomato fruits showed the accelarated ripening phenomenon. We also used the ethylene receptor mutant *Nr* and several ethylene signaling defective mutants, *rin, nor*, and *Cnr*, to determine the functions of the OGs on the ethylene signaling pathway. None of the mutant fruits’ ripening processes were accelerated by OGs. Therefore, we demonstrated that the OGs’ functions on fruit ripening required ethylene signaling pathway and the autocatalytic regulation of ethylene synthesis. Our study also revealed that the transcriptional levels of *LeACS2* and *LeACO1* were rapidly up-regulated in the presence of OGs. Moreover, OGs could induce the phosphorylation of LeACS2 at Ser-460. These results demonstrated that OGs induced MG 1 tomato fruit ethylene biosynthesis at the transcriptional and post-translational levels, and then promoted the ripening of tomato fruits. Additionally, we found a candidate receptor of the OGs, LeWAKL2, which was also induced by the OGs at the gene and protein levels.

## Additional files

Additional file 1:
**Ethylene production of AC fruits at MG 2/3 stages after treatment.** Tomato fruits were placed in a ventilated and temperature constant room at 25 °C and treated with 1 g/L OGs or the control solution. Ethylene production was detected every day after treatment. Vertical bars indicate the *SD* (*n* = 6). (PDF 89 kb) Additional file 2:
**Long-term ethylene production of** ***rin*** **,** ***nor*** **and** ***Cnr*** **tomato fruits after treatment.** Tomato fruits were placed in a ventilated and temperature constant room at 25 °C and treated with 1 g/L OGs or the control solution. Ethylene production was detected every day after treatment. Vertical bars indicate the *SD* (*n* = 6). (PDF 93 kb) Additional file 3:
**Transient ethylene production of AC and mutant fruits pericarp discs after treatment.** 1 mL gas was extracted to detect ethylene content. Vertical bars indicate the *SD* (*n* = 4), asterisks indicate statistically significant differences compared with control group (**P* < 0.05; ***P* < 0.01, Student’s *t*-test). (PDF 105 kb) Additional file 4:
**Separation and characterization of individual OGs.** (**A**) Uronic acids content of separation products, every three tube were chosen to be detected use microplate reader, thirteen individual peaks were found. (**B**) TLC experiment was developed to detect the degree of polymerization of OGs in the peak, nine distinct points were observed according to the DP from 2 to 10. (**C**) MALDI-MS was used to testify the contents of separation products, OGs with a DP of 4 was shown, m/z = 745.1 was the right format of GalA_4_-Na^+^. (PDF 195 kb) Additional file 5:
**Ethylene production of AC fruits treated with different OG mixtures.** Four groups were: control, mixed OG with DP < 9, mixed OG with DP ≥ 9, mixed OG with all the DP. The total mass concentration of each mixture were adjusted to 1 g/L. Ethylene productions were detected every day after treatment. Vertical bars indicate the *SD* (*n* = 6), significant differences of four groups at each time point were indicated by different letters (Tukey’s HSD, *P* < 0.05). (PDF 189 kb) Additional file 6:
**Primer sequences used for RT-PCR.** (PDF 146 kb) Additional file 7:
**Gene IDs used in this study.** (PDF 4 kb)

## Abbreviations

ABA: Abscisic acid
AC: Ailsa Craig
ACC: 1-aminocyclopropane-1-carboxylic acid
ACO: ACC oxidase
ACS: ACC synthesis
Cnr: Colorless non-ripening
DAP: Days after pollination
DMSO: Dimethylsulfoxide
DP: Degree of polymerization
GC: Gas chromatograph
MALDI-MS: Matrix-assisted laser desorption ionization-mass spectrum
MG: Mature green
nor: Non-ripening
Nr: Neverripe
OA: Okadaic acid
OGs: Oligogalacturonic acids
PG: Polygalacturonase
PGA: Polygalacturonic acid
PME: Pectin methylesterase
rin: Ripening-inhibitor
TLC: Thin-layer chromatography
WAK/WAKL: Wall associated kinase/kinase like
