# Supplementary figures and images for: Oligogalacturonic acids promote tomato fruit ripening through the regulation of 1-aminocyclopropane-1-carboxylic acid synthesis at the transcriptional and post-translational levels
Source: BMC Plant Biol. 2016 Jan 9;16:13. doi: 10.1186/s12870-015-0634-y (PMC4706653; doi:10.1186/s12870-015-0634-y)

Additional file 1: Ethylene production of AC fruits at MG 2/3 stages after treatment.

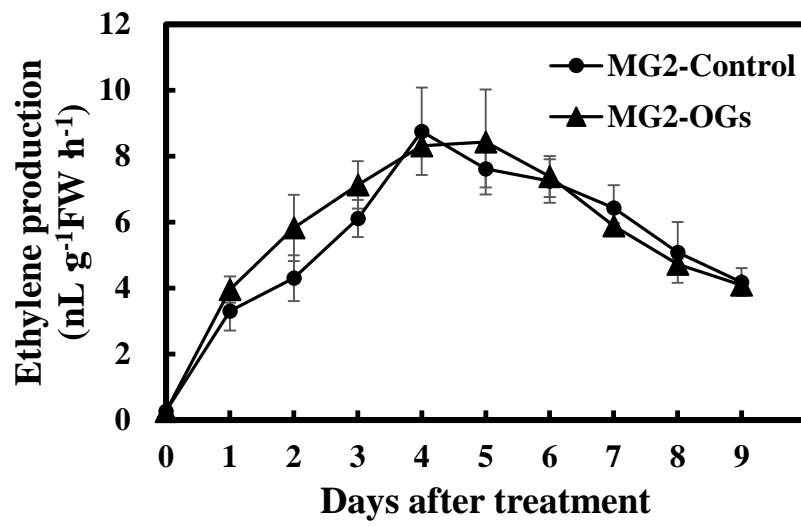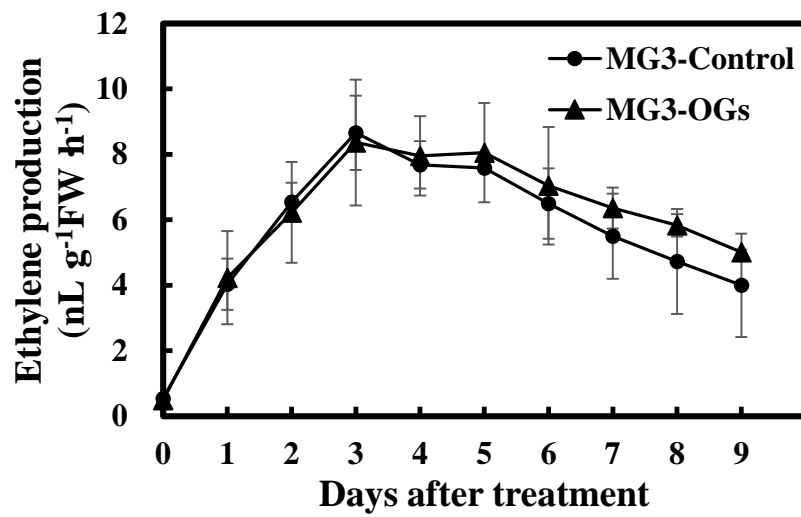

Supplement: Additional file 1: — Ethylene production of AC fruits at MG 2/3 stages after treatment. Tomato fruits were placed in a ventilated and temperature constant room at 25 °C and treated with 1 g/L OGs or the control solution. Ethylene production was detected every day after treatment. Vertical bars indicate the SD (n = 6). (PDF 89 kb) [file 12870_2015_634_MOESM1_ESM.pdf]

Additional file 4: Separation and characterization of individual OGs.

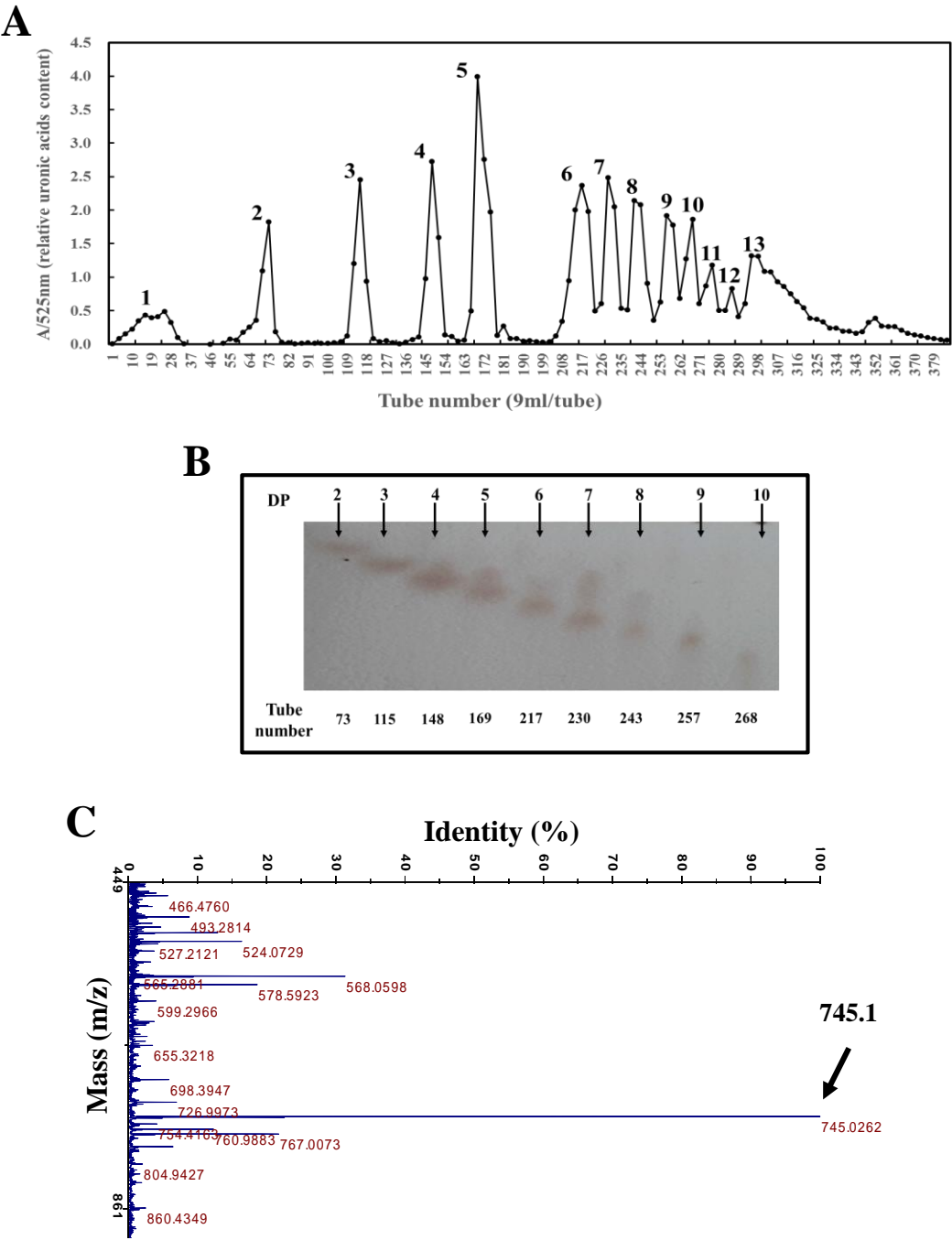

Supplement: Additional file 4: — Separation and characterization of individual OGs. (A) Uronic acids content of separation products, every three tube were chosen to be detected use microplate reader, thirteen individual peaks were found. (B) TLC experiment was developed to detect the degree of polymerization of OGs in the peak, nine distinct points were observed according to the DP from 2 to 10. (C) MALDI-MS was used to testify the contents of separation products, OGs with a DP of 4 was shown, m/z = 745.1 was the right format of GalA4-Na+. (PDF 195 kb) [file 12870_2015_634_MOESM4_ESM.pdf]

Additional file 5: Ethylene production of AC fruits treated with different OG mixture.

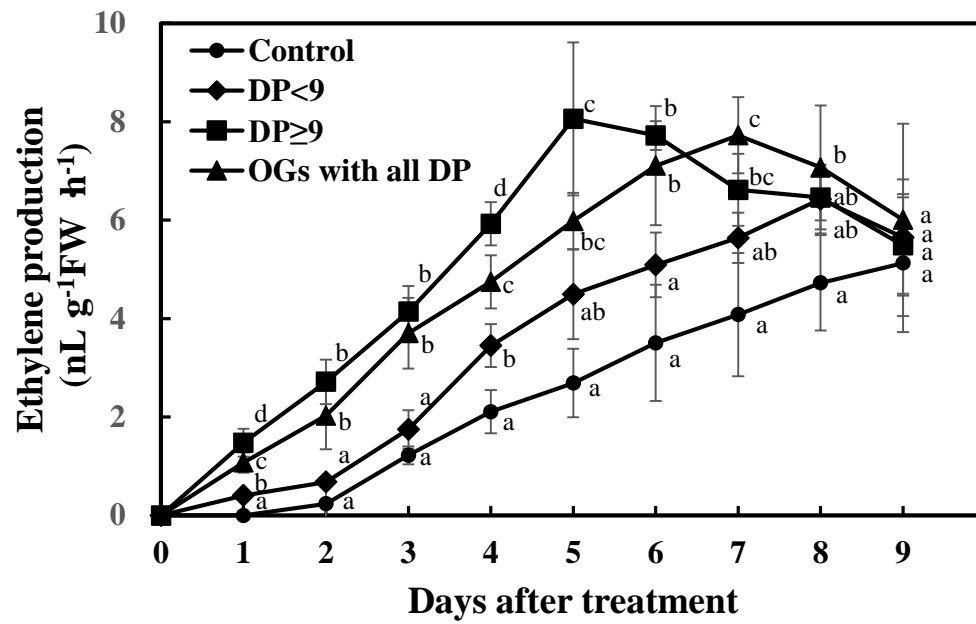

Supplement: Additional file 5: — Ethylene production of AC fruits treated with different OG mixtures. Four groups were: control, mixed OG with DP < 9, mixed OG with DP ≥ 9, mixed OG with all the DP. The total mass concentration of each mixture were adjusted to 1 g/L. Ethylene productions were detected every day after treatment. Vertical bars indicate the SD (n = 6), significant differences of four groups at each time point were indicated by different letters (Tukey’s HSD, P < 0.05). (PDF 189 kb) [file 12870_2015_634_MOESM5_ESM.pdf]
