# Supplementary material for: Oligogalacturonic acids promote tomato fruit ripening through the regulation of 1-aminocyclopropane-1-carboxylic acid synthesis at the transcriptional and post-translational levels
Source: BMC Plant Biol. 2016 Jan 9;16:13. doi: 10.1186/s12870-015-0634-y (PMC4706653; doi:10.1186/s12870-015-0634-y)

Additional file 2: Long-term ethylene production of *rin*, *nor* and *Cnr* tomato fruits after treatment.

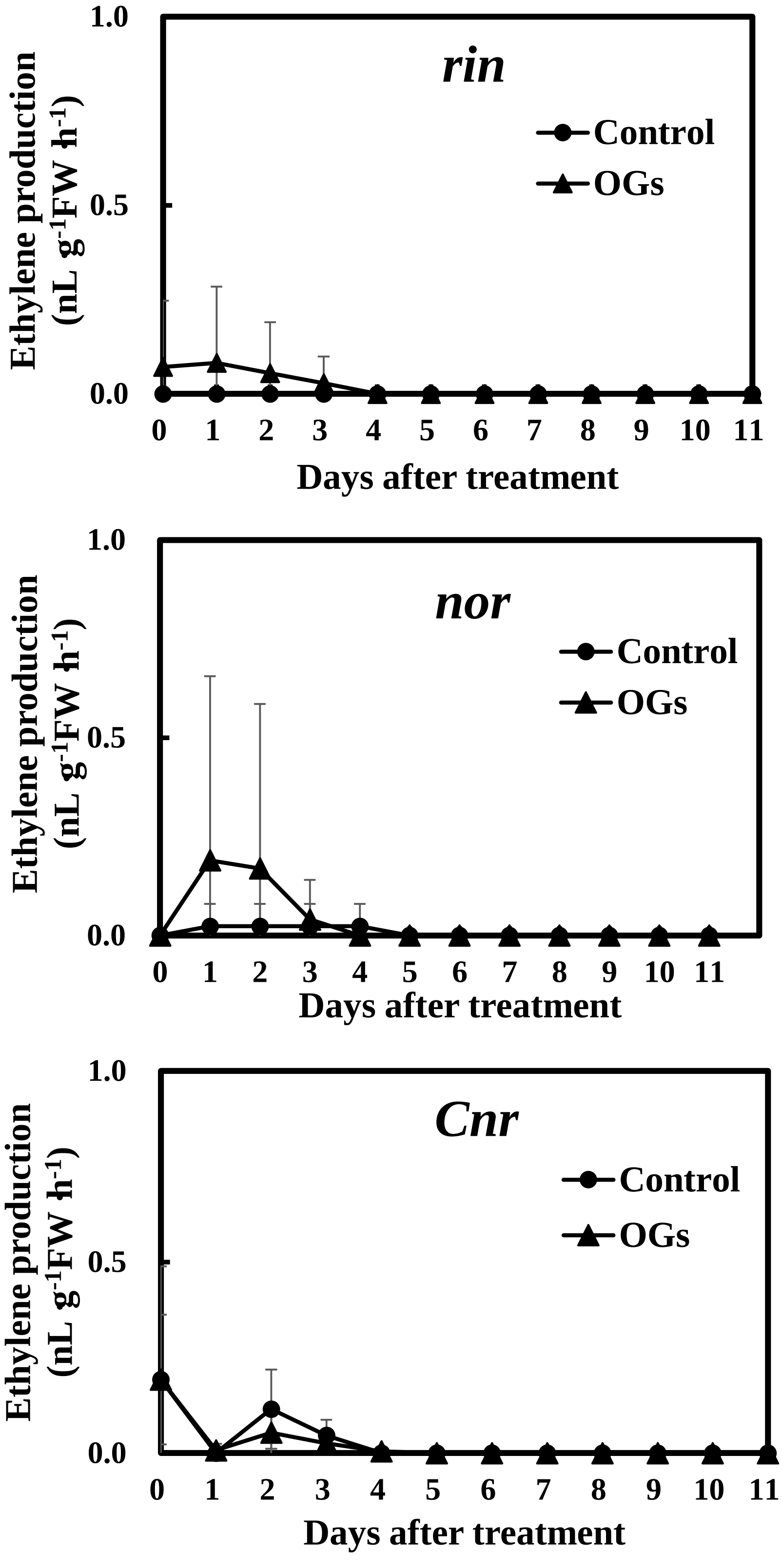

Supplement: Additional file 2: — Long-term ethylene production of rin , nor and Cnr tomato fruits after treatment. Tomato fruits were placed in a ventilated and temperature constant room at 25 °C and treated with 1 g/L OGs or the control solution. Ethylene production was detected every day after treatment. Vertical bars indicate the SD (n = 6). (PDF 93 kb) [file 12870_2015_634_MOESM2_ESM.pdf]
