# Supplementary material for: Oligogalacturonic acids promote tomato fruit ripening through the regulation of 1-aminocyclopropane-1-carboxylic acid synthesis at the transcriptional and post-translational levels
Source: BMC Plant Biol. 2016 Jan 9;16:13. doi: 10.1186/s12870-015-0634-y (PMC4706653; doi:10.1186/s12870-015-0634-y)
Supplement: Additional file 3: — Transient ethylene production of AC and mutant fruits pericarp discs after treatment. 1 mL gas was extracted to detect ethylene content. Vertical bars indicate the SD (n = 4), asterisks indicate statistically significant differences compared with control group (*P < 0.05; **P < 0.01, Student’s t-test). (PDF 105 kb) [file 12870_2015_634_MOESM3_ESM.pdf]

Additional file 3: Transient ethylene production of AC and mutant fruits pericarp discs after treatment.

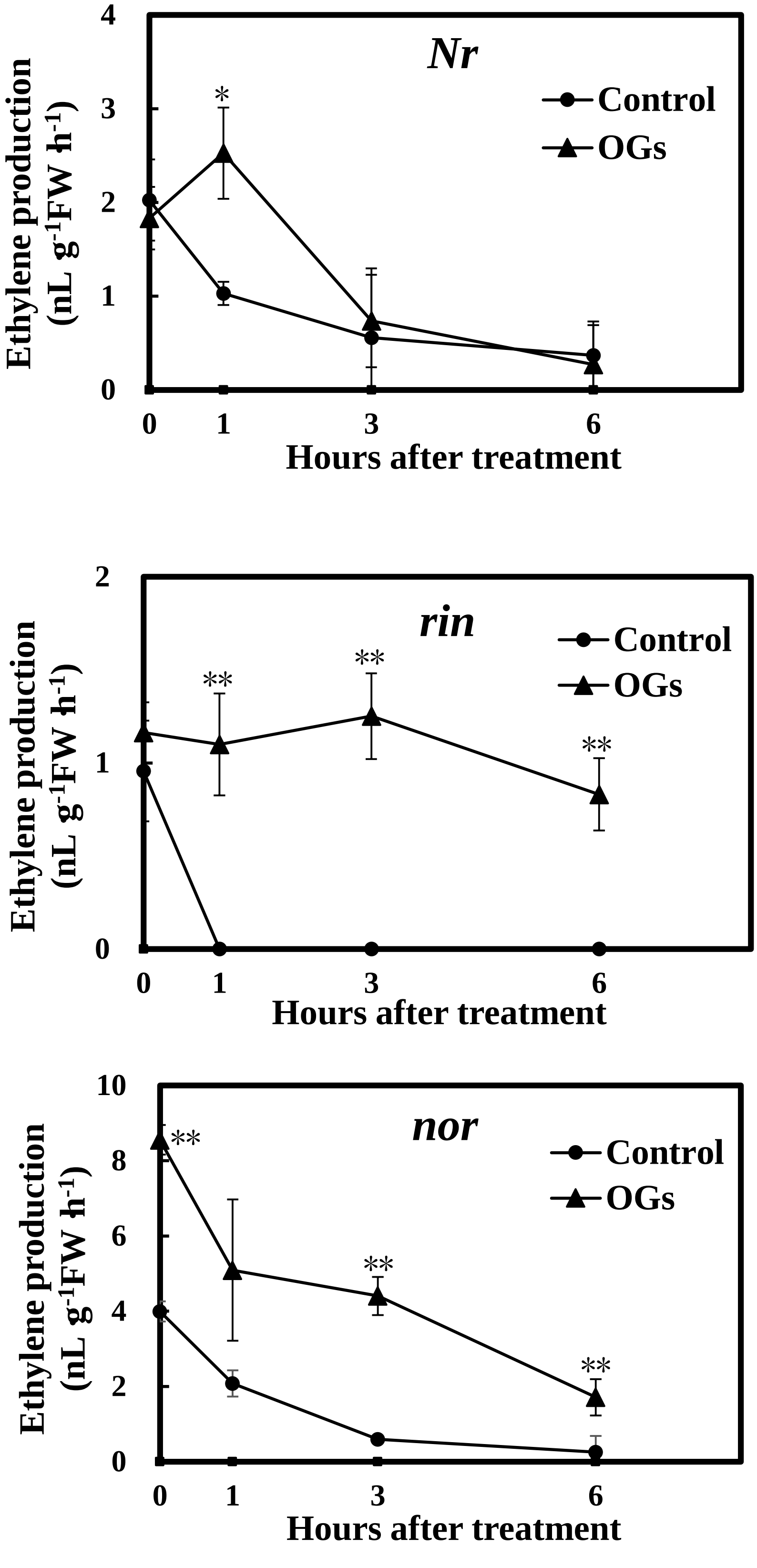

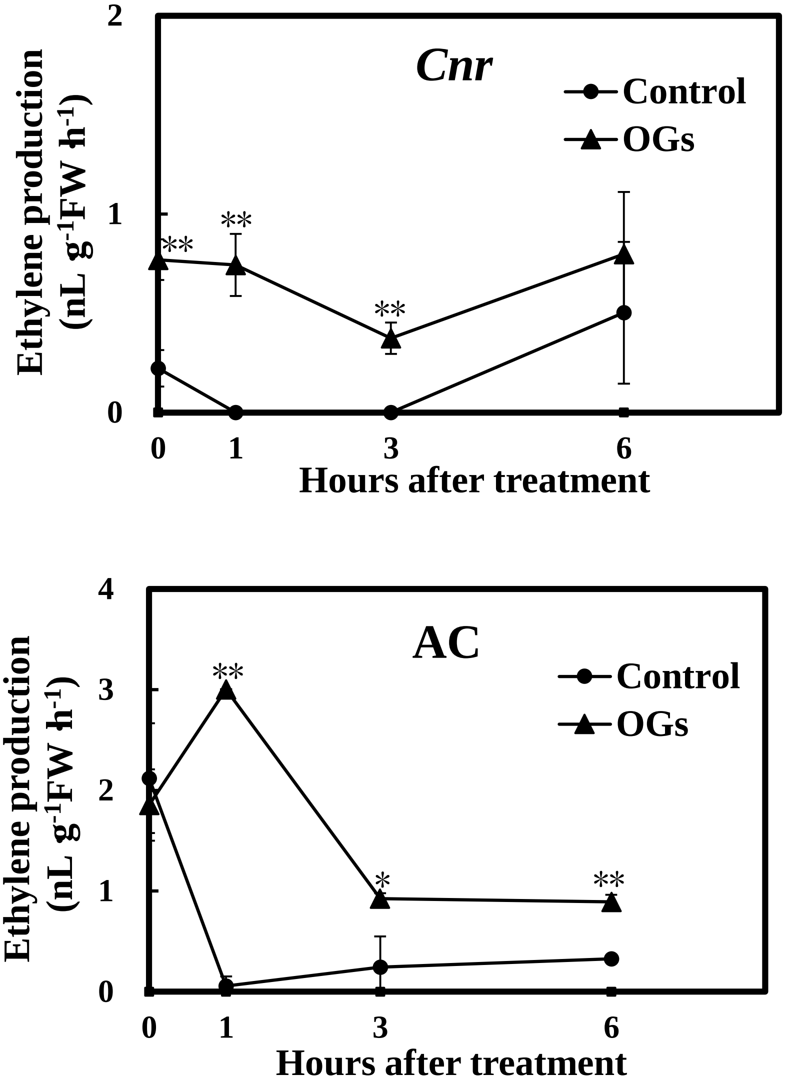

Cnr
Control
OGs
Ethylene production (nL g-1FW h-1)
Hours after treatment
AC
Control
OGs
Ethylene production (nL g-1FW h-1)
Hours after treatment
