# Supplementary material for: Oligogalacturonic acids promote tomato fruit ripening through the regulation of 1-aminocyclopropane-1-carboxylic acid synthesis at the transcriptional and post-translational levels
Source: BMC Plant Biol. 2016 Jan 9;16:13. doi: 10.1186/s12870-015-0634-y (PMC4706653; doi:10.1186/s12870-015-0634-y)
Supplement: Additional file 6: — Primer sequences used for RT-PCR. (PDF 146 kb) [file 12870_2015_634_MOESM6_ESM.pdf]

Primers for RT-PCR

Gene name	Forward primer	Reverse primer
Actin	cagcagatgtggatctcaaa	ctgtggacaatggaaggac
LeACS1A	tgtttcaccaggatgttcatttc	cgaaacgataagctgatctgaagt
LeACS2	tggatgatggaacggttgatattgc	ccattgttgcttctgttccatcgaac
LeACS4	gatcttcgttcaattgctcggaggt	gcatccataaacatccgaatccttg
LeACS6	cctggttgttcatttcattgctcagag	gcaacttcaactcccttatttggttgtaa
LeACO1	cttgaggtgatcactaacgggaagtaca	tgctggatatattactgcatcacttcctg
LeWAKL2	ttgggctaatggacatggac	tcaccgcggaatatttgtctt
