# Supplementary material for: Oligogalacturonic acids promote tomato fruit ripening through the regulation of 1-aminocyclopropane-1-carboxylic acid synthesis at the transcriptional and post-translational levels
Source: BMC Plant Biol. 2016 Jan 9;16:13. doi: 10.1186/s12870-015-0634-y (PMC4706653; doi:10.1186/s12870-015-0634-y)
Supplement: Additional file 7: — Gene IDs used in this study. (PDF 4 kb) [file 12870_2015_634_MOESM7_ESM.pdf]

Additional file 7: Gene IDs used in this study.

LeActin (LOC101264601), LeACS1A (U72389.1), LeACS2 (NM_001247249.1), LeACS4 (NM_001247351.1), LeACS6 (AF179249.1), LeACO1 (NM_001247095.1) mRNA sequences were downloaded from National Center for Biotechnology Information (NCBI), LeWAKL2 (Solyc02g090110.2.1) sequence was downloaded from Sol Genomics Network.
